# Threshold effects of atherogenic index of plasma on female infertility risk: evidence from NHANES 2013–2018

**DOI:** 10.1186/s12905-025-04128-w

**Published:** 2025-11-19

**Authors:** Hongtao Zhu, Luqiong Gong, Huihua Yan

**Affiliations:** Department of Obstetrics and Gynecology, The Affiliated Yangming Hospital of Ningbo University, No. 800, Chengdond Road, Yuyao, Ningbo, Zhejiang Province 315400 China

**Keywords:** Atherogenic index of plasma, Female infertility, Lipid metabolism, NHANES, Risk assessment

## Abstract

**Background:**

The relationship between lipid metabolism and female infertility remains incompletely understood. The Atherogenic Index of Plasma (AIP) has emerged as a valuable marker of metabolic dysfunction, yet its association with infertility risk has not been systematically investigated in a large population-based study.

**Methods:**

Data from 3,454 reproductive-aged women were analyzed using the National Health and Nutrition Examination Survey (NHANES) (2013–2018). Statistical analyses incorporated NHANES sampling weights. The association between AIP and infertility was examined using weighted logistic regression models with progressive adjustment for demographic and lifestyle factors (age, race, education, poverty income ratio, smoking, alcohol consumption, BMI, and diabetes status). Restricted cubic spline and threshold effect analyses (a statistical approach to identify inflection points where the strength of association changes) were performed to explore potential non-linear relationships and inflection points.

**Results:**

Higher AIP levels were independently associated with increased infertility risk (adjusted OR = 2.292, 95% CI: 1.414–3.714, *P* = 0.001). This association demonstrated significant age modification, with stronger effects observed in women under 30 years (OR = 5.258, 95% CI: 2.054–13.455, *P* = 0.001). Exploratory threshold effect analysis suggested a potential inflection point at AIP = -0.076, below which the association was particularly pronounced (OR = 4.365, 95% CI: 2.002–9.863, *P* < 0.001), though the likelihood ratio test did not reach conventional statistical significance (*P* = 0.074).

**Conclusion:**

AIP shows promise as a biomarker for female infertility risk assessment, particularly in younger women. The identified AIP threshold of -0.076 is preliminary and hypothesis-generating, requiring external validation before clinical implementation. These findings suggest potential utility for risk stratification and warrant further prospective investigation to establish its clinical utility.

## Introduction

Infertility represents the inability to achieve pregnancy despite 12 months of regular unprotected intercourse [1]. Recent global epidemiological data indicate that infertility affects an estimated 186 million individuals worldwide [2]. Longitudinal epidemiological studies demonstrate a concerning trend, with female-specific infertility prevalence rates increasing by 0.370% annually from 1990 to 2017^3^. While infertility typically manifests with limited physical symptomatology, research indicates that women facing this condition commonly report mental health challenges including heightened anxiety levels, depressive symptoms, and diminished overall well-being [4, 5]. At the interpersonal level, infertility frequently compromises relationship dynamics, contributing to decreased marital satisfaction and increased rates of marital dissolution [6–8]. These multifaceted impacts, encompassing individual psychological burden, social consequences, and substantial healthcare expenditures [9], establish infertility as a significant public health challenge that necessitates comprehensive research and evidence-based therapeutic interventions.

Extensive research has established multiple etiological factors contributing to female infertility, encompassing age-related decline, immunological dysfunction, endocrine disorders, anatomical reproductive abnormalities, and modifiable lifestyle determinants [10–12]. Among these factors, advanced maternal age emerges as a paramount determinant, with clinical evidence demonstrating a marked deterioration in reproductive potential beyond age 35^13^. In recent years, metabolic dysregulation, particularly perturbations in lipid metabolism, has emerged as a significant contributor to female reproductive dysfunction [14, 15]. Contemporary scientific investigations have elucidated several mechanistic pathways through which altered lipid profiles impact reproductive function, including endocrine disruption [16], enhanced oxidative stress [17], and aberrant inflammatory cascades [18].

Clinical observations reveal that women with dysregulated lipid metabolism commonly manifest impaired reproductive outcomes, characterized by compromised oocyte quality, aberrant embryonic development, and reduced implantation efficiency [19, 20]. These metabolic perturbations demonstrate particularly strong associations with polycystic ovary syndrome [21], a predominant etiology of female infertility. However, current investigative approaches examining the intersection of lipid metabolism and reproductive dysfunction face significant methodological limitations. Standard lipid panels yield limited pathophysiological understanding, and body mass index (BMI) measurements insufficiently reflect fat distribution patterns and metabolic status [22]. This analytical gap necessitates more refined biomarkers for assessing lipid metabolism’s influence on reproductive function.

Derived from the logarithmic ratio of triglycerides (TG) to high-density lipoprotein cholesterol (HDL-C) levels [23], Atherogenic Index of Plasma (AIP) serves as an advanced indicator of lipoprotein dynamics. This measurement offers unique insights into metabolic health by assessing the relationship between harmful and protective lipid particles, providing greater diagnostic precision than standard lipid testing methods. The clinical utility of AIP demonstrates notable advantages over traditional lipid assessments [24], particularly in its methodological simplicity, requiring only standard lipid profile measurements, thus ensuring both economic efficiency and broad clinical implementation potential.

Substantial clinical evidence has established AIP’s robust predictive capacity across various metabolic disorders, encompassing both cardiovascular pathologies [25] and diabetic complications [26]. Of particular relevance to reproductive medicine, research has demonstrated significant correlations between elevated AIP values and two critical pathophysiological mechanisms: insulin resistance [27] and enhanced oxidative stress [28], both of which exert effects on reproductive function [29]. These established associations, combined with its comprehensive metabolic assessment capabilities, position AIP as a potentially valuable biomarker for investigating the complex interrelationships between lipid metabolism and female reproductive potential.

Despite established links between lipid metabolism and reproduction, along with AIP’s proven value in metabolic assessment, studies examining AIP’s impact on fertility outcomes remain limited. This knowledge gap assumes critical significance given that current diagnostic protocols may inadequately evaluate the metabolic components underlying infertility, potentially resulting in delayed therapeutic interventions and suboptimal personalized treatment strategies.

Using National Health and Nutrition Examination Survey (NHANES) data collected between 2013 and 2018, we examined the potential correlation between AIP values and reproductive outcomes in a broad population-based analysis. Our research hypothesis posits that AIP measurements may serve as effective indicators of infertility risk, providing clinicians with an accessible, cost-effective screening tool for reproductive health assessment.

## Methods

### Study design

NHANES operates as a national survey system that evaluates the health and nutrition status of the U.S. population using standardized epidemiological methods. This research initiative employs sophisticated multistage probability sampling techniques to collect detailed participant data through structured interviews, standardized clinical examinations, and comprehensive biochemical analyses. The National Center for Health Statistics Ethics Review Board approved this research, with documented informed consent obtained from all participants.

**Fig. 1:**
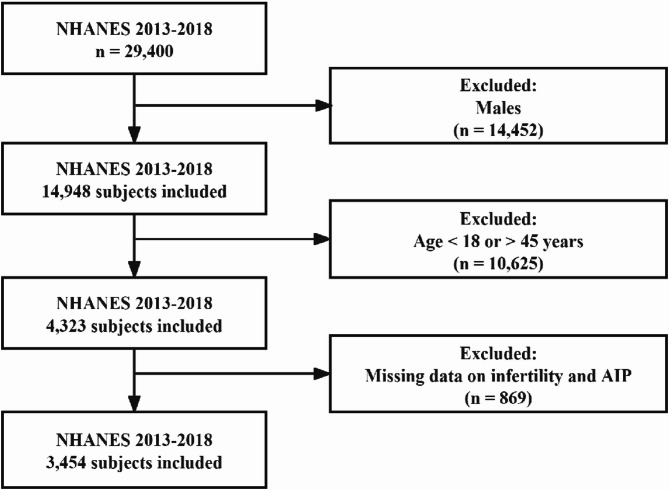
Study Population Selection Framework

Our investigation analyzed data encompassing three consecutive NHANES cycles from 2013 to 2018. The participant selection followed a systematic approach, beginning with an initial population of 29,400 (Fig. 1) individuals. To establish a focused study cohort relevant to female reproductive health, we first excluded male participants (*n* = 14,452). From the remaining female population (*n* = 14,948), we selected participants within reproductive age parameters (18–45 years), excluding 10,625 individuals who fell outside this age range [30]. This initial screening yielded 4,323 eligible participants. Following a thorough assessment of data completeness, we further excluded 869 participants with incomplete infertility status documentation or missing AIP measurements, resulting in a final analytical cohort of 3,454 women who fulfilled all predefined inclusion criteria.

### Definition of infertility

Infertility assessment was conducted using standardized protocols through the NHANES reproductive health questionnaire administered at designated Mobile Examination Centers via the Computer Assisted Personal Interview system. The primary diagnostic criterion for infertility followed standard clinical protocols, characterizing the condition as unsuccessful conception following one year of regular reproductive attempts. Assessment was specifically based on responses to questionnaire item RHQ074, which asked: “Have you ever attempted to become pregnant over a period of at least a year without becoming pregnant?” Participants responding “yes” were classified as having experienced infertility, while those responding “no” were classified as fertile [31, 32]. To maintain methodological integrity and minimize potential classification bias, we systematically excluded participants who provided indeterminate responses (including “don’t know” responses) or those who declined to respond to the fertility assessment questions. This operational definition aligns with standard clinical criteria for infertility and has been consistently applied in previous NHANES-based reproductive health studies.

### Definition of AIP

AIP values were derived from comprehensive biochemical measurements obtained through standardized NHANES laboratory protocols. All participants were required to fast for at least 8 h prior to blood collection, and fasting status was verified before sample collection. The index calculation followed established methodology, employing a mathematical transformation based on the logarithmic relationship between key lipid parameters. The AIP was calculated as log10[TG/HDL-C], where TG and HDL-C were measured in millimoles per liter (mmol/L) [33].

### Covariates

Our multivariate analysis incorporated demographic and behavioral covariates to account for potential confounding effects. The demographic variables included age, race/ethnicity, educational level, and socioeconomic status. We stratified socioeconomic status using the poverty income ratio (PIR), which compares household income to federal poverty thresholds. Lifestyle factors were systematically evaluated through standardized assessments of tobacco use and alcohol consumption patterns. Smoking status classification utilized a cumulative exposure criterion, with participants reporting lifetime consumption of ≥ 100 cigarettes designated as smokers. Regular alcohol use was defined as more than 12 drinking occasions in the past year. Participants not meeting these predefined thresholds were classified as non-smokers and non-drinkers, respectively. Body mass index (BMI) was calculated as weight in kilograms divided by height in meters squared (kg/m²). Diabetes status was defined based on self-reported physician diagnosis.

### Statistical analysis

All analyses were performed in R 4.3.1 incorporating NHANES sampling weights. Continuous variables were reported as median (interquartile range) and categorical variables as weighted counts (%). Group comparisons used weighted Wilcoxon rank-sum and chi-square tests for continuous and categorical variables, respectively.

The AIP-infertility association was assessed through three weighted logistic regression models: unadjusted (Model 1), adjusted for demographics (Model 2: age, race, education, PIR), and fully adjusted (Model 3: adding smoking and alcohol use). Forest plots displayed odds ratios with 95% confidence intervals. Subgroup analyses were conducted to explore potential effect modifications, with interaction terms tested in the fully adjusted model. Non-linear relationships were evaluated using restricted cubic spline (RCS) with five knots placed at the 5th, 27.5th, 50th, 72.5th, and 95th percentiles of AIP distribution, following Harrell’s recommendations for balancing model flexibility and stability, and two-piecewise linear regression for threshold effects. Three sensitivity analyses were performed to test the robustness of our findings. Model 3 A additionally adjusted for BMI and diabetes status. While BMI is associated with both dyslipidemia and infertility risk, it may also lie on the causal pathway between metabolic dysfunction and reproductive outcomes. To address this complexity, we examined BMI adjustment in a sensitivity analysis rather than the primary model. This approach allows assessment of whether AIP provides metabolic information relevant to infertility beyond general adiposity. Model 3B used multiple imputation with chained equations to handle missing covariate data. Model 3 C excluded currently pregnant women to eliminate potential confounding by pregnancy status. Statistical significance was set at *P* < 0.05.

## Results

### Baseline characteristics of study participants

Our study analysis, incorporating NHANES sampling weights, represented approximately 50.2 million U.S. women (unweighted *n* = 3,454). Demographic and metabolic characteristics differed markedly between fertility groups, as detailed in Table 1. The infertile cohort demonstrated significantly advanced age compared to their fertile counterparts (median age 36.00 versus 31.00, *P* < 0.001) and exhibited a higher proportion of smoking behavior (37.74% versus 30.03%, *P* = 0.009).

**Table 1:** Baseline characteristics between infertility and fertility groups

Variables	Total *N* = 3454 Weighted *N* = 50,186,414	Fertility *N* = 3085 Weighted *N* = 44,289,447	Infertility *N* = 369 *N* = 5,896,967	*P*
Age (median [IQR])	31.00 [25.00, 39.00]	31.00 [24.00, 38.00]	36.00 [30.00, 41.00]	< 0.001
Race (%)				0.182
Mexican American	6109490.14 (12.17)	5512309.95 (12.45)	597180.19 (10.13)	
Other Hispanic	4024959.90 (8.02)	3641176.09 (8.22)	383783.82 (6.51)	
Non-Hispanic White	28275145.39 (56.34)	24599174.30 (55.54)	3675971.09 (62.34)	
Non-Hispanic Black	6447174.47 (12.85)	5730960.57 (12.94)	716213.90 (12.15)	
Other Race	5329644.63 (10.62)	4805826.61 (10.85)	523818.02 (8.88)	
Education (%)				0.673
Less Than 9th Grade	1582090.05 (3.36)	1457660.98 (3.54)	124429.07 (2.12)	
9-11th Grade	3912957.51 (8.32)	3395182.39 (8.25)	517775.12 (8.83)	
High School Grad	9016110.19 (19.17)	7867134.68 (19.11)	1148975.52 (19.60)	
Some College or AA degree	17299721.41 (36.78)	15186529.91 (36.88)	2113191.50 (36.05)	
College Graduate or above	15229278.93 (32.38)	13271831.16 (32.23)	1957447.77 (33.39)	
PIR (%)				0.403
≤ 1.3	13706400.12 (29.35)	12255726.88 (29.84)	1450673.24 (25.73)	
> 1.3 and ≤ 3.5	17229978.60 (36.89)	15083746.70 (36.73)	2146231.91 (38.06)	
> 3.5	15769091.52 (33.76)	13727421.03 (33.43)	2041670.49 (36.21)	
Smoking status (%)				0.009
No	34650437.86 (69.07)	30979119.86 (69.97)	3671318.00 (62.26)	
Yes	15519457.07 (30.93)	13293808.05 (30.03)	2225649.02 (37.74)	
Alcohol drinking status (%)				0.979
No	16486400.07 (33.85)	14533377.51 (33.86)	1953022.56 (33.78)	
Yes	32215499.60 (66.15)	28387037.55 (66.14)	3828462.05 (66.22)	
TG (median [IQR])	1.07 [0.74, 1.59]	1.04 [0.73, 1.56]	1.26 [0.84, 1.88]	< 0.001
HDL-C (median [IQR])	1.42 [1.19, 1.71]	1.45 [1.19, 1.71]	1.34 [1.09, 1.63]	0.008
BMI (median [IQR])	27.50 [22.90, 33.80]	27.10 [22.70, 33.20]	31.00 [24.80, 37.44]	< 0.001
Diabetes (%)				< 0.001
No	47653069.96 (96.37)	42362338.19 (96.98)	5290731.77 (91.74)	
Yes	1795781.09 (3.63)	1319225.64 (3.02)	476555.45 (8.26)	
AIP (median [IQR])	−0.14 [−0.33, 0.10]	−0.15 [−0.34, 0.08]	−0.05 [−0.25, 0.19]	< 0.001
AIP quartile (%)				< 0.001
Q1	12537983.46 (24.98)	11656352.95 (26.32)	881630.51 (14.95)	
Q2	12725325.15 (25.36)	11370150.15 (25.67)	1355175.00 (22.98)	
Q3	12875317.63 (25.65)	11153315.06 (25.18)	1722002.58 (29.20)	
Q4	12047788.28 (24.01)	10109629.35 (22.83)	1938158.93 (32.87)	

The most striking distinctions emerged in metabolic parameters. Women with infertility displayed substantially altered lipid profiles, characterized by elevated triglyceride concentrations (1.26 versus 1.04, *P* < 0.001) and decreased HDL-C levels (1.34 versus 1.45, *P* = 0.008). These lipid alterations manifested in significantly higher AIP values among infertile women (−0.05 versus − 0.15, *P* < 0.001). Furthermore, the distribution of AIP quartiles also differed significantly between groups (*P* < 0.001), with infertile women showing higher proportions in the upper quartiles.

The overall prevalence of infertility in our study population was 11.75%. Infertility prevalence demonstrated a clear gradient across AIP quartiles: Q1 (lowest AIP) = 7.03%, Q2 = 10.65%, Q3 = 13.37%, and Q4 (highest AIP) = 16.09%. This represents a 2.3-fold increase in absolute risk from the lowest to highest AIP quartile, with an absolute risk difference of 9.06% points.

### Association between AIP and infertility risk

**Fig. 2:**
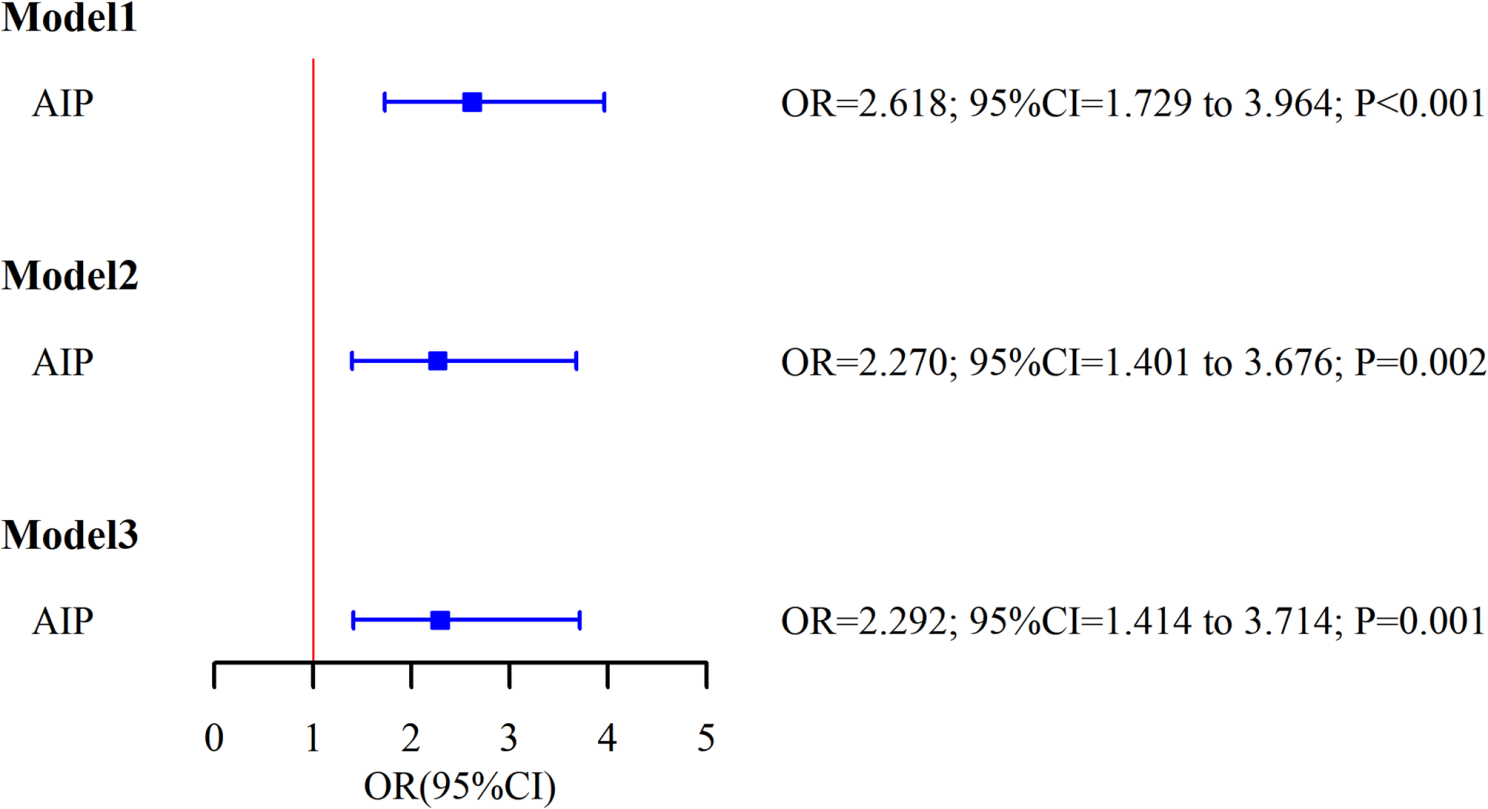
Forest plot of logistic regression

Statistical modeling demonstrated a consistent association between AIP and infertility risk across three sequentially adjusted models (Table 2; Fig. 2). The elevated risk of infertility with increasing AIP values persisted after adjusting for potential demographic, socioeconomic, and behavioral confounding variables. Model 3 quantified that a one-unit elevation in AIP was associated with a 129.2% increased odds of infertility after controlling for all covariates (OR = 2.292, 95% CI: 1.414–3.714, *P* = 0.001).

**Table 2:** Logistic regression results: AIP and infertility risk

	Model 1		Model 2		Model 3	
	OR(95% CI)	*P*	OR(95% CI)	*P*	OR(95% CI)	*P*
AIP	2.618 (1.729, 3.964)	< 0.001	2.270 (1.401, 3.676)	0.002	2.292 (1.414, 3.714)	0.001
AIP quartile						
Q 1	Reference	/	Reference	/	Reference	/
Q 2	1.576 (1.060, 2.342)	0.026	1.553 (1.025, 2.353)	0.039	1.658 (1.072, 2.564)	0.025
Q 3	2.041 (1.397, 2.982)	< 0.001	2.012 (1.318, 3.071)	0.002	2.127 (1.371, 3.298)	0.001
Q 4	2.535 (1.656, 3.88)	< 0.001	2.246 (1.398, 3.610)	0.002	2.350 (1.453, 3.801)	0.001
*P* for trend		< 0.001		0.001		< 0.001

Further analysis by AIP quartiles demonstrated a clear dose-dependent relationship with infertility risk. Subjects in the uppermost AIP quartile demonstrated significantly elevated infertility odds relative to those in the lowest quartile, with an adjusted OR of 2.350 (95% CI: 1.453–3.801, *P* = 0.001). Trend analysis across quartiles yielded a P-value < 0.001, substantiating a robust positive correlation between ascending AIP levels and infertility risk.

### Subgroup analysis

The subgroup analyses (Table 3) identified a significant effect modification by age (*P* interaction = 0.015). The AIP-infertility association was markedly stronger in women under 30 years of age (OR = 5.258, 95% CI: 2.054–13.455, *P* = 0.001) compared with those 30 years or older (OR = 1.981, 95% CI: 1.152–3.407, *P* = 0.015). However, the wide confidence interval for the younger age group warrants cautious interpretation of this finding.

**Table 3:** Subgroup analysis of AIP-Infertility associations

Subgroup	OR(95% CI)	*P*	*P* for interaction
Overall	2.292 (1.414, 3.714)	0.001	
Age			0.015
< 30	5.258 (2.054, 13.455)	0.001	
≥ 30	1.981 (1.152, 3.407)	0.015	
Race (%)			0.105
Mexican American	3.382 (1.484, 7.711)	0.006	
Other Hispanic	0.859 (0.171, 4.304)	0.846	
Non-Hispanic White	2.786 (1.325, 5.857)	0.008	
Non-Hispanic Black	3.675 (1.484, 9.099)	0.007	
Other Race	0.551 (0.266, 1.14)	0.105	
Smoking status (%)			0.658
No	2.101 (1.179, 3.747)	0.013	
Yes	2.667 (1.339, 5.313)	0.007	
Alcohol drinking status (%)			0.107
No	1.383 (0.563, 3.397)	0.467	
Yes	2.890 (1.689, 4.946)	< 0.001	

Regarding racial/ethnic groups, significant associations were observed among Mexican Americans (*P* = 0.006), non-Hispanic Whites (*P* = 0.008), and non-Hispanic Blacks (*P* = 0.007), although the interaction test was not significant (*P* for interaction = 0.105).

The relationship remained significant regardless of smoking status, with similar associations observed in both smokers (*P* = 0.007) and non-smokers (*P* = 0.013). Among alcohol users, the association was significant only in those who reported drinking (*P* < 0.001), though the interaction test was not significant (*P* for interaction = 0.107).

### Non-linear association analysis

**Fig. 3:**
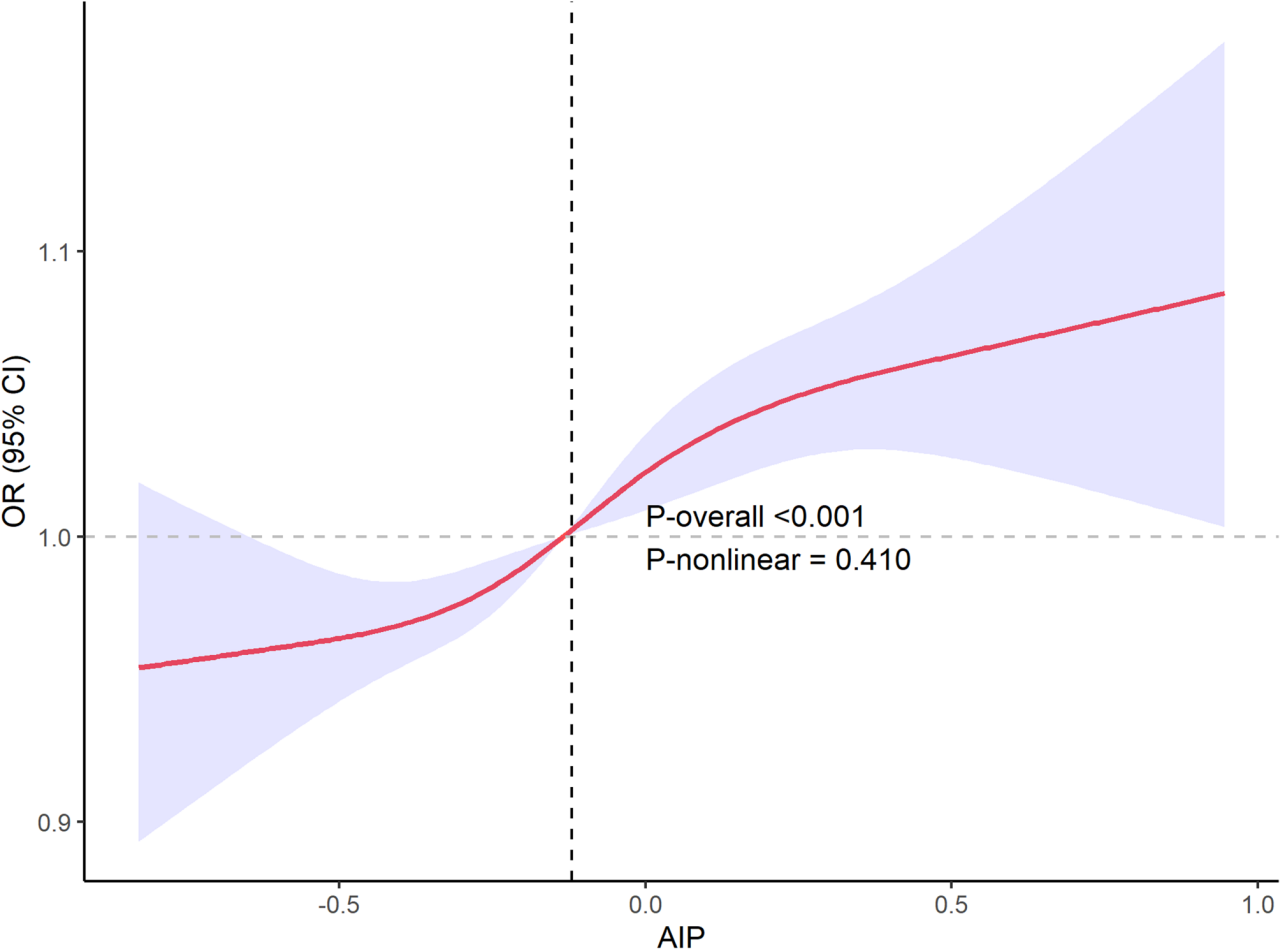
AIP-Infertility Risk Restricted Cubic Spline Analysis

Restricted cubic spline analysis with five knots demonstrated a strong overall relationship between AIP and infertility (*P*-overall < 0.001), but revealed no significant non-linear patterns (*P*-nonlinear = 0.410). The association maintained linearity across the observed AIP range (Fig. 3). The vertical dashed line indicates the potential inflection point at AIP = −0.076 identified in exploratory threshold analysis.

### Threshold effect analysis

To complement our RCS analysis, threshold analysis using two-piecewise linear regression was performed to examine potential inflection points where the AIP-infertility relationship changes (Table 4). This analysis suggested a possible inflection point at AIP = −0.076, with distinct patterns of association above and below this value. For AIP values below − 0.076, we observed a strong positive association with infertility risk (OR = 4.365, 95% CI: 2.002–9.863, *P* < 0.001). Above the suggested threshold of −0.076, the AIP-infertility association weakened considerably (OR = 1.528, 95% CI: 0.865–2.640, *P* = 0.135). Likelihood ratio testing compared the two-piecewise and single linear models to validate this threshold effect. The test result (*P* = 0.074) approached but did not reach conventional statistical significance, indicating that this finding should be interpreted as exploratory rather than conclusive evidence of a biological threshold. This potential inflection point warrants further investigation in future prospective studies.

**Table 4:** AIP threshold analysis and infertility

		OR(95% CI)	*P*
Fitting by standard linear model	AIP	2.285(1.655–3.148)	< 0.001
Fitting by two-piecewise linear model	Inflection point	−0.076	
	AIP < −0.076	4.365(2.002–9.863)	< 0.001
	AIP > −0.076	1.528(0.865–2.640)	0.135
	*P* for likelihood ratio test		0.074

### Sensitivity analyses

Three sensitivity analyses were performed to assess the robustness of our findings (Table 5). After additional adjustment for BMI and diabetes status (Model 3 A), the positive association between AIP and infertility remained significant (OR = 2.222, 95% CI: 1.376–3.588, *P* = 0.002), indicating that the AIP-infertility association is independent of general adiposity. The dose-response relationship across AIP quartiles was also maintained (*P* for trend < 0.001), with the highest quartile showing more than two-fold increased odds compared to the lowest quartile (OR = 2.345, 95% CI: 1.468–3.745, *P* = 0.001).

**Table 5:** Sensitivity analyses of the association between AIP and infertility risk

	Model 3 A		Model 3B		Model 3 C	
	OR(95% CI)	*P*	OR(95% CI)	*P*	OR(95% CI)	*P*
AIP	2.222 (1.376, 3.588)	0.002	2.079 (1.329, 3.253)	0.001	2.183 (1.359, 3.508)	0.002
AIP quartile						
Q 1	Reference	/	Reference	/	Reference	/
Q 2	1.768 (1.172, 2.668)	0.008	1.526 (1.034, 2.254)	0.033	1.703 (1.11, 2.612)	0.017
Q 3	2.290 (1.523, 3.444)	< 0.001	1.967 (1.344, 2.878)	0.001	2.374 (1.58, 3.566)	< 0.001
Q 4	2.345 (1.468, 3.745)	0.001	2.172 (1.396, 3.381)	0.001	2.257 (1.441, 3.534)	0.001
*P* for trend		< 0.001		< 0.001		< 0.001

Model 3 A: Model 3 with additional adjustment for BMI and diabetes status

Model 3B: Model 3 using multiple imputation for missing covariates

Model 3 C: Model 3 after excluding currently pregnant women

Multiple imputation analysis for missing covariate data (Model 3B) yielded consistent results with the complete case analysis (OR = 2.079, 95% CI: 1.329–3.253, *P* = 0.001), suggesting minimal impact of missing data on our findings.

After excluding currently pregnant women (Model 3 C), the AIP-infertility association remained robust (OR = 2.183, 95% CI: 1.359–3.508, *P* = 0.002), confirming that pregnancy status did not substantially confound our results. All three sensitivity analyses demonstrated consistent dose-response patterns across AIP quartiles, supporting the stability and reliability of our primary findings.

## Discussion

This NHANES-based study represents the first comprehensive analysis examining AIP as a potential biomarker for female infertility risk in a nationally representative population. Statistical analysis demonstrated that elevated AIP values independently predicted increased infertility risk (OR = 2.292, 95% CI: 1.414–3.714). Quantile analysis revealed a dose-response relationship, as participants in the highest AIP quartile exhibited a 2.35-fold greater infertility risk compared to those in the lowest quartile (OR = 2.350, 95% CI: 1.453–3.801). Notably, stratification by age identified a pronounced effect modification, with the AIP-infertility association showing particular strength in women below 30 years of age (OR = 5.258, 95% CI: 2.054–13.455). While this age-stratified analysis included a substantial sample size (unweighted *n* = 1,633), the wide confidence interval suggests this finding should be interpreted as preliminary evidence requiring validation in future prospective studies. This age-dependent effect suggests that lipid metabolism may exert particularly strong influences on fertility during early reproductive years. Further analysis suggested a potential AIP threshold at −0.076, below which the association with infertility was especially pronounced (OR = 4.365, 95% CI: 2.002–9.863), although the likelihood ratio test (*P* = 0.074) indicates this should be considered a hypothesis-generating finding rather than definitive evidence of a biological threshold. The identification of both age-specific effects and a potential threshold value provides preliminary insights that may inform future research on reproductive health screening and intervention strategies.

It is important to acknowledge the apparent discordance between our restricted cubic spline analysis and threshold analysis results. The RCS analysis indicated an overall linear relationship between AIP and infertility risk (*P*-nonlinear = 0.410), while the threshold analysis suggested a potential inflection point at AIP = −0.076, though this did not reach conventional statistical significance (*P* = 0.074). These seemingly inconsistent findings warrant careful interpretation. The two analytical approaches examine different aspects of the AIP-infertility relationship: RCS analysis tests for smooth, continuous non-linear patterns across the entire range of observed AIP values, whereas threshold analysis specifically examines whether distinct linear relationships exist above and below a certain inflection point. The absence of significant non-linearity in the RCS analysis, combined with the suggestive but non-significant threshold effect, may reflect several possibilities. First, the relationship between AIP and infertility may be predominantly linear across most of the AIP distribution, with any threshold effect being subtle or confined to specific ranges. Second, our sample size, while substantial, may have limited power to definitively detect and confirm a true threshold effect if one exists. Third, the biological relationship may indeed be linear rather than threshold-based, and the observed inflection point in the threshold analysis could represent random variation.

Several biological mechanisms may explain the potential threshold pattern observed in our exploratory analysis. In the higher AIP range (above approximately − 0.08, near the population median), most women may already have established metabolic dysfunction with multiple risk factors for infertility. In this context, further increases in AIP may not substantially add to infertility risk, as the metabolic damage is already present. Conversely, the transition from very low AIP values (e.g., −0.3, indicating favorable lipid profiles) to moderate levels (around − 0.08) may represent the onset of clinically significant metabolic disruption. This transition could mark the point at which atherogenic dyslipidemia begins to exert meaningful effects on reproductive function through mechanisms such as insulin resistance, oxidative stress, and inflammatory processes. This post-hoc biological interpretation remains speculative and requires validation through mechanistic studies and prospective investigations that can establish temporal relationships between AIP changes and fertility outcomes. Given these uncertainties, we present both analyses transparently and emphasize that the potential threshold at −0.076 should be considered hypothesis-generating rather than definitive. Future studies with larger sample sizes and longitudinal designs are needed to clarify the precise nature of the dose-response relationship between AIP and infertility risk.

The robust association between AIP and female infertility identified in our study can be understood through multiple interrelated pathophysiological pathways. While our cross-sectional design cannot establish causality, several mechanistic pathways may explain the observed association. This index provides more comprehensive metabolic insights than conventional lipid parameters, particularly in assessing cardiometabolic risk [34, 35]. Research has demonstrated AIP’s superior capacity to characterize atherogenic lipoprotein profiles, specifically in reflecting particle size and density distributions that may not be captured by standard lipid measurements [36]. This enhanced diagnostic precision is particularly relevant given that both key components of AIP - elevated TG levels and reduced HDL-C concentrations - have independently demonstrated associations with compromised reproductive outcomes [37]. The integration of these parameters into a single index may therefore provide more nuanced insights into the metabolic perturbations affecting fertility.

A primary mechanism connecting elevated AIP to infertility operates through insulin resistance and associated metabolic dysregulation. Research has established AIP as an effective surrogate indicator of insulin resistance, with elevated AIP values demonstrating strong inverse correlations with insulin sensitivity [38]. This relationship has particular significance in reproductive health, as insulin resistance disrupts normal reproductive function through several well-documented pathways. Insulin resistance disrupts normal hypothalamic-pituitary-gonadal signaling [39], resulting in irregular gonadotropin secretion and impaired follicular development that compromises fertility [40]. Furthermore, the compensatory hyperinsulinemia that accompanies insulin resistance stimulates excessive androgen production in ovarian theca cells [41]. This pathophysiological cascade has particular relevance in polycystic ovary syndrome, where metabolic dysfunction often underlies reproductive impairment [42].

The interaction between AIP and oxidative stress represents another critical mechanism influencing fertility outcomes. Studies have shown that higher AIP levels correlate with both elevated oxidative stress biomarkers and compromised antioxidant defenses [43]. This relationship has substantial implications for reproductive function through multiple cellular and molecular pathways. Oxidative stress particularly affects oocyte quality and maturation through disruption of mitochondrial function. This perturbation leads to compromised ATP production and impairment of essential cellular processes necessary for successful fertilization [44]. At the cellular level, oxidative damage to lipids and proteins initiates apoptotic cascades in granulosa cells, consequently affecting both follicular development and steroidogenic function [45]. Additionally, oxidative stress impacts endometrial tissue, where it can compromise endometrial receptivity and interfere with early embryonic development [46]. The prominent role of oxidative stress may help explain our observation of stronger AIP-infertility associations in younger women. We hypothesize that metabolic-induced oxidative stress may exert more pronounced effects on reproductive function before age-related factors become predominant. This age-dependent effect suggests that addressing metabolic health early in reproductive life may be particularly beneficial for fertility outcomes. However, given the limited precision of our age-stratified estimates, this hypothesis requires confirmation in larger studies of younger women before definitive conclusions can be drawn about addressing metabolic health early in reproductive life as a fertility preservation strategy.

A critical methodological consideration in our analysis is the relationship between AIP, adiposity, and infertility. Our sensitivity analysis adjusting for BMI and diabetes status (Model 3 A) demonstrated that the AIP-infertility association remained significant (OR = 2.222, 95% CI: 1.376–3.588, *P* = 0.002), indicating that AIP captures metabolic information relevant to fertility beyond what is reflected by general adiposity. The slight attenuation of the effect size compared to the primary model suggests that adiposity accounts for some, but not all, of the observed association. This finding is important because it suggests that atherogenic dyslipidemia—characterized by the specific combination of elevated triglycerides and reduced HDL-C that AIP quantifies—may influence fertility through mechanisms partially independent of total body fat. While BMI reflects overall adiposity, AIP may better capture the specific lipid perturbations, insulin resistance, and oxidative stress that directly impact reproductive function. This distinction has clinical relevance, as it suggests that metabolic interventions targeting lipid profiles may benefit fertility even in women without obesity, and conversely, that weight management alone may be insufficient for women with atherogenic dyslipidemia.

Our findings offer several valuable insights that may, pending validation, inform future approaches to fertility risk assessment and management. The emergence of AIP as a potential biomarker for reproductive health holds particular promise due to its practical advantages in clinical settings. The absolute prevalence data demonstrate clinically meaningful risk differences, with infertility prevalence ranging from 7.03% in the lowest AIP quartile to 16.09% in the highest quartile. This 2.3-fold increase in absolute risk (9.06% point difference) suggests that AIP could potentially help identify women at elevated fertility risk, though prospective validation is needed to confirm its predictive utility in clinical practice.The AIP calculation leverages routine lipid panel measurements, and our sensitivity analysis demonstrates that it provides information relevant to fertility beyond what is captured by BMI alone. This suggests it represents a cost-effective screening tool that integrates seamlessly into existing clinical workflows. Exploratory threshold analysis suggested a potential inflection point at AIP = −0.076, below which infertility risk was particularly elevated (OR = 4.365, 95% CI: 2.002–9.863). However, given that the likelihood ratio test did not reach conventional statistical significance (*P* = 0.074), this threshold should not be applied in clinical practice until confirmed by additional research. If validated in future prospective studies, such a threshold could provide clinicians with a concrete reference point for risk stratification.

The observed age-dependent relationship between AIP and infertility necessitates a more nuanced approach to fertility assessment. Our findings suggest, though require prospective validation, that notably stronger association among women under 30 years may indicate that early metabolic screening could be particularly valuable for identifying fertility risks in younger populations. However, this hypothesis requires testing in prospective studies before any screening recommendations can be made. This finding has important implications for the timing of interventions, potentially allowing for earlier identification and management of fertility challenges before they become more difficult to address.

Our results also inform preventive medicine and lifestyle modification strategies. The modifiable nature of lipid profiles through lifestyle interventions presents an opportunity for proactive fertility management. Studies have indicated that structured lifestyle adjustments, such as adopting healthier dietary patterns and engaging in regular exercise, can effectively improve both metabolic parameters and fertility outcomes [47, 48]. However, whether lowering AIP through lifestyle modifications, pharmacological interventions, or other means directly improves fertility outcomes remains to be established. Intervention studies are needed to determine whether targeting AIP reduction translates into meaningful improvements in conception rates. Early identification of metabolic risk factors through AIP screening could facilitate timely lifestyle interventions, potentially preserving natural fertility and reducing reliance on costly assisted reproductive technologies.

### Strengths and limitations

The use of nationally representative NHANES data provides a robust foundation for generalizing our results to the broader population. We employed rigorous statistical methodologies, including regression modeling with adjustment for demographic and lifestyle factors. The application of advanced analytical techniques, such as threshold effect analysis and restricted cubic spline analysis, enabled us to identify both age-specific interactions and a clinically meaningful AIP threshold value.

The cross-sectional study design precludes determination of temporal associations between AIP and infertility, thereby limiting causal inference. Women who never attempted pregnancy were classified as “fertile” by default, which may not reflect their actual fertility potential. This misclassification, if non-differential with respect to AIP levels, would likely bias our results toward the null. Our analysis could not distinguish between female factor, male factor, or combined infertility, and since infertility represents a couple-level outcome, our findings reflect associations with couple-level infertility rather than isolated female reproductive dysfunction. We could not differentiate between specific infertility etiologies, which may have different relationships with metabolic markers.

Additionally, infertility status was ascertained through self-reported questionnaire data rather than clinical confirmation, which may introduce recall bias and potential misclassification. Participants may have varying interpretations of their fertility attempts or inaccurately recall the duration, potentially leading to non-differential misclassification that could attenuate the observed associations. The exclusion of 869 participants with incomplete data could introduce selection bias if these individuals systematically differed from the analytic sample (e.g., women with missing lipid data may have been more likely to be pregnant or have other conditions). Furthermore, the NHANES question captures a history of 12-month infertility but does not indicate whether this was subsequently resolved or whether participants eventually conceived, limiting direct comparability to clinical infertility diagnoses. Generalizability to non-U.S. populations requires validation, as differences in genetic backgrounds, lifestyle factors, and healthcare systems may affect the AIP-infertility relationship.

Furthermore, several important infertility-related factors were not available in the NHANES dataset, including parity, contraceptive use history, and specific diagnoses of polycystic ovary syndrome (PCOS) and thyroid disorders. The absence of these variables represents potential residual confounding that may partially explain the observed associations. While sensitivity analyses adjusting for BMI and diabetes showed consistent results, the lack of PCOS and thyroid disorder information is particularly notable given their strong associations with both metabolic dysfunction and infertility risk.

These limitations illuminate several critical directions for future research. Future longitudinal investigations are needed to delineate the temporal relationship between AIP and infertility. More granular investigations examining AIP’s relationship with specific infertility types would enhance our understanding and inform targeted therapeutic approaches. Intervention studies assessing whether improvements in AIP values translate to enhanced fertility outcomes would provide valuable clinical evidence. Additionally, validation studies across diverse populations are needed to confirm the broader applicability of our findings and solidify AIP’s role as a clinical marker for fertility risk assessment.

## Conclusions

Our study provides evidence for a significant association between the AIP and female infertility risk. Exploratory threshold analysis suggested a possible AIP threshold value of −0.076 represents a potentially valuable clinical benchmark, as the relationship with infertility risk was particularly pronounced below this level. However, as the likelihood ratio test did not reach conventional statistical significance (*P* = 0.074), this finding should be considered hypothesis-generating rather than definitive. If validated in future prospective studies, this potential threshold could serve as a clinical reference point for risk stratification.

The notably stronger association observed among women under 30 years of age suggests that AIP screening may be especially beneficial during early reproductive years, though this finding requires confirmation in future prospective studies given the wide confidence intervals observed in this age subgroup. These findings advance our understanding of lipid metabolism’s role in reproductive health in two important ways. First, they establish AIP as a promising clinical marker for early identification of fertility risks, particularly in younger women. Second, they identify a potential threshold value that, if confirmed in prospective research, could potentially be used by clinicians for risk stratification. AIP evaluation requires only standard lipid profiles, rendering it a cost-effective tool for reproductive health screening. While our findings provide a foundation for future research, they also establish a clear pathway for additional prospective studies to further validate AIP’s utility in fertility assessment and management.

## Data Availability

All study data are available for download on the NHANES website (https://www.cdc.gov/nchs/nhanes/).
